# A systematic review of financial toxicity measurement instruments for cancer patients based on the COSMIN guideline

**DOI:** 10.1186/s41687-026-01042-z

**Published:** 2026-03-19

**Authors:** Mingfang Jia, Liyi Zhong, Yingxia Luo, Oudong Xia

**Affiliations:** 1 School of Health Management, Southern Medical University, Guangzhou, China; 2 Department of Nursing, Zhujiang Hospital of Southern Medical University, Guangzhou, China; 3 Shantou University Medical College, Shantou, China

**Keywords:** Financial toxicity, Cancer patients, PROMs, COSMIN, Systematic review

## Abstract

**Objective:**

Financial toxicity, characterized by the financial burden and psychological distress associated with cancer treatment, has become a critical issue impacting patient outcomes and quality of life. This study aimed to conduct an updated and comprehensive systematic review to evaluate the methodological quality and measurement properties of financial toxicity measurement instruments for cancer patients, thereby guiding the selection of high-quality tools for future empirical research.

**Methods:**

Literature on financial toxicity measurement instruments for cancer patients was retrieved from CNKI, Wanfang, VIP, SinoMed, PubMed, Web of Science, CINAHL, Scopus, and Embase databases, with a search range from the inception up to April 16, 2025. Two researchers independently screened the literature and extracted data. The included studies were assessed using the Consensus-Based Standards for the Selection of Health Measurement Instruments (COSMIN), and recommendations were formulated based on the quality of evidence and measurement properties.

**Results:**

A total of eighteen studies were included, involving eight financial toxicity measurement instruments for cancer patients. None of the studies reported measurement error. The Comprehensive Score for Financial Toxicity (COST), Financial Index of Toxicity (FIT), and Subjective Financial Distress Questionnaire (SFDQ) were provisionally recommended for use (Category B), while the other five instruments were not recommended due to insufficient evidence (Category C).

**Conclusion:**

The COST is provisionally recommended for measuring financial toxicity in cancer patients. The methodological quality and measurement properties of existing financial toxicity instruments still require further improvement. Future research should adhere strictly to the COSMIN guideline to validate existing tools or develop new, high-quality instruments with comprehensive evaluations of their measurement properties.

## Introduction

Cancer is one of the most severe global health challenges, with 20 million new cases reported worldwide in 2022 [1]. At the same time, significant advancements in cancer treatment technology have markedly prolonged the survival of cancer patients, contributing to a rapidly expanding global population of cancer survivors—with over 18.6 million survivors in the U.S. alone as of January 1, 2025, projected to exceed 22 million by 2035 [2]. Nevertheless, the associated high medical expenses, including both direct medical expenses such as treatments, medications and indirect costs such as travel, lost income for patients and caregivers, impose a significant financial burden on patients, their families, and healthcare systems. This negative financial impact, termed ‘financial toxicity’, has become an irremediable side effect of cancer treatment [3]. Financial toxicity is a multidimensional construct encompassing both objective economic burdens and subjective financial distress experienced by cancer patients due to treatment [4]. It manifests in three core domains: one is the material domain, characterized by substantial out-of-pocket expenditures, depletion of savings, debt accumulation, and asset liquidation; the second is the psychological domain, exemplified by stress, anxiety, and depression caused by medical expenses; and the third is the behavioral dimension, evidenced by coping behaviors such as delaying or abandoning necessary treatment [5].

Even in countries with well-established healthcare systems, financial toxicity remains prevalent. For example, in the U.S., the incidence of financial toxicity among cancer patients is as high as 55% under the mixed payment system, while in the UK, which has a publicly funded health system, this rate drops to 34%, yet over one-third of cancer patients still face financial toxicity [6]. In low- and middle-income countries, financial toxicity is even more severe. Out-of-pocket expenses of cancer patients are significantly higher than in high-income countries [7], frequently triggering catastrophic medical expenditures. This perpetuates a vicious cycle of ‘poverty due to illness and abandonment of treatment due to poverty’. Studies have shown that financial toxicity greatly reduces cancer patients’ quality of life and treatment compliance of cancer patients, increasing psychological stress and ultimately affecting survival outcomes [3, 8–10].

To measure and intervene against financial toxicity, researchers have developed and validated multiple Patient-Reported Outcome Measures (PROMs), including the COST [11, 12], Patient-Reported Outcome for Fighting Financial Toxicity (PROFFIT) [13], and Financial Index of Toxicity (FIT) [14]. These PROMs have performed well in preliminary psychometric properties. However, as highlighted in previous systematic reviews [15–17] and conceptual analyses [4, 5], they still have the following limitations: (1) there is limited evidence in the psychometric domains of cross-cultural validity, measurement error, and responsiveness, which are crucial for ensuring an instrument’s validity across diverse populations and its ability to detect change over time; (2) there is incomplete conceptual coverage. While financial toxicity is recognized as a multidimensional construct, most PROMs rely heavily on subjective perceptions, and the integration of objective indicators remains inconsistent, leading to variations in what is being measured; (3) the field lacks unified standards, with large discrepancies between PROMs in defining and operationalising financial toxicity, making it difficult to compare study results horizontally. These limitations hinder their clinical utility and necessitate a systematic evaluation based on international standards. The COSMIN guideline is an internationally recognized standard for selecting health measurement instruments [18]. It provides a methodological framework for systematic reviews of PROMs. Central to this framework is the hierarchical evaluation of nine measurement properties, with content validity considered the most important. The guideline employs a structured, step-by-step process to evaluate measurement properties, combined with a modified GRADE approach to synthesize evidence and grade recommendations. This rigorous methodology ensures the scientific rigor and clinical applicability of the resulting recommendations. While previous systematic reviews have assessed psychometric properties of financial toxicity measures in cancer patients [15, 19, 20], this review aims to provide an updated and comprehensive evaluation based on the latest COSMIN guideline [18]. This allows for a more current and rigorous comparison of available tools and provides clearer, evidence-based recommendations for their use in research and clinical practice. This systematic review was conducted in accordance with the COSMIN methodology for systematic reviews and the Reporting Items for Systematic Reviews and Meta-Analyses statement. It has been registered in PROSPERO (registration number: CRD420251061606).

## Methods

### Inclusion and exclusion criteria

The inclusion criteria were as follows: (1) studies that targeted cancer patients who were ≥ 18 years old; (2) studies that aimed to develop or validate a financial toxicity measurement instrument reported and assessed at least one measurement property; (3) studies published in English or Chinese. The exclusion criteria were as follows: (1) studies that used a PROM only as an outcome measurement; (2) secondary literature, such as reviews and meta-analyses; (3) studies for which the full text was not available; (4) studies that were duplicates.

### Search strategy

A literature search was conducted in nine databases, including CNKI, Wanfang, VIP, SinoMed, PubMed, Web of Science, CINAHL, Scopus, and Embase. The search was conducted from the inception of each database up to April 16, 2025. The English search terms included ‘neoplasm/tumor/cancer/malignancy’, ‘economic burden/financial toxicity/financial hardship’, ‘scale/questionnaire/instrument’, ‘reliability/validity’. In PubMed, the search strategy was as follows: ((“neoplasms”[MeSH Terms] OR tumor*[Title/Abstract] OR cancer*[Title/Abstract]) AND (“cancer survivors”[Title/Abstract] OR patient*[Title/Abstract] OR survivor*[Title/Abstract]) AND (“cost of illness”[Title/Abstract] OR cost*[Title/Abstract] OR bill*[Title/Abstract] OR expense*[Title/Abstract] OR “productivity loss”[Title/Abstract] OR “out-of-pocket”[Title/Abstract] OR “economic burden”[Title/Abstract] OR “financial toxicity”[Title/Abstract] OR “financial hardship”[Title/Abstract]) AND (“scale”[Title/Abstract] OR measure*[Title/Abstract] OR “patient-reported outcome measure*”[Title/Abstract]) AND (psychometric*[Title/Abstract] OR “measurement properties”[Title/Abstract] OR validity[Title/Abstract] OR reliability[Title/Abstract] OR “internal consistency”[Title/Abstract])).

### Study screening and data extraction

Two researchers independently screened the literature and extracted data. The extracted information included the authors, publication year, instrument name, country, sample size, participants, domains and items, total score range, scoring method, completion time, retest interval, and measurement property results.

### Assessment procedure

**Fig. 1:**
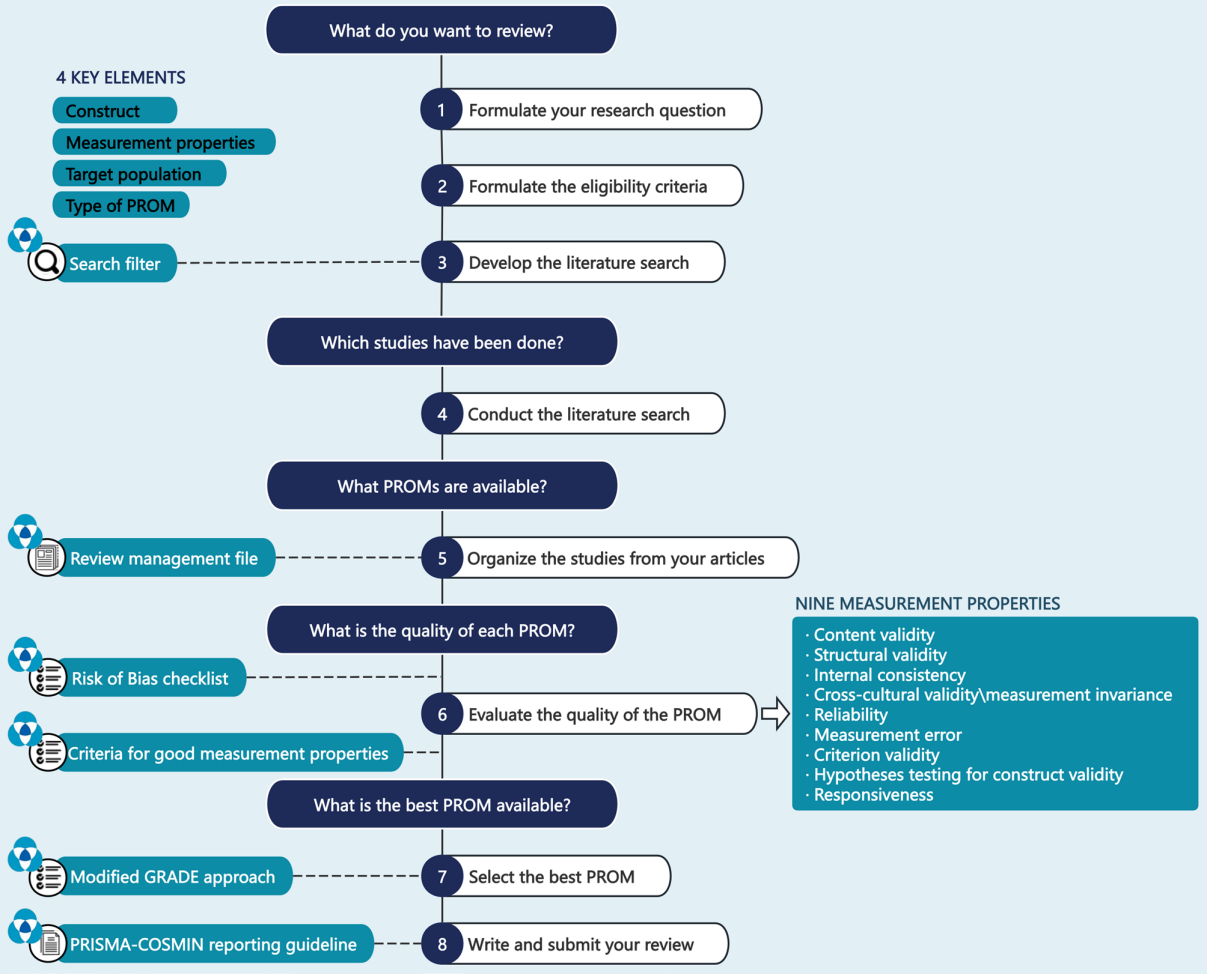
Eight steps for conducting a systematic review of PROMs. (adapted from [18])

Two researchers independently assessed the methodological quality, measurement properties, and certainty of evidence of the included studies according to the COSMIN guideline. The overall process of conducting a systematic review of PROMs is shown in Fig. 1 (adapted from [18]). Disagreements were resolved by a third researcher, and final recommendations were formulated.

### Assessment methodology

#### Assessment of methodological quality

The methodological quality of the included studies was assessed using the COSMIN Risk of Bias Checklist version 3 [18]. It comprises PROM development, content validity, structural validity, internal consistency, cross-cultural validity/measurement invariance, reliability, measurement error, criterion validity, hypotheses testing for construct validity, and responsiveness. Each item scored on a four-point scale, ranging from ‘very good’ to ‘inadequate’. The overall rating of the quality of each study was determined by the lowest rating of any standard in the checklist, following the ‘worst score counts’ principle [21].

#### Assessment of measurement properties

The nine measurement properties of the PROMs were evaluated against the COSMIN criteria for good measurement properties version 2 [18]. It included content validity, structural validity, internal consistency, cross-cultural validity/measurement invariance, reliability, measurement error, criterion validity, hypotheses testing for construct validity, and responsiveness. Each measurement property from each study was rated as ‘sufficient (+)’, ‘insufficient (–)’, or ‘indeterminate (?)’.

#### Grading the certainty of evidence

**Fig. 2:**
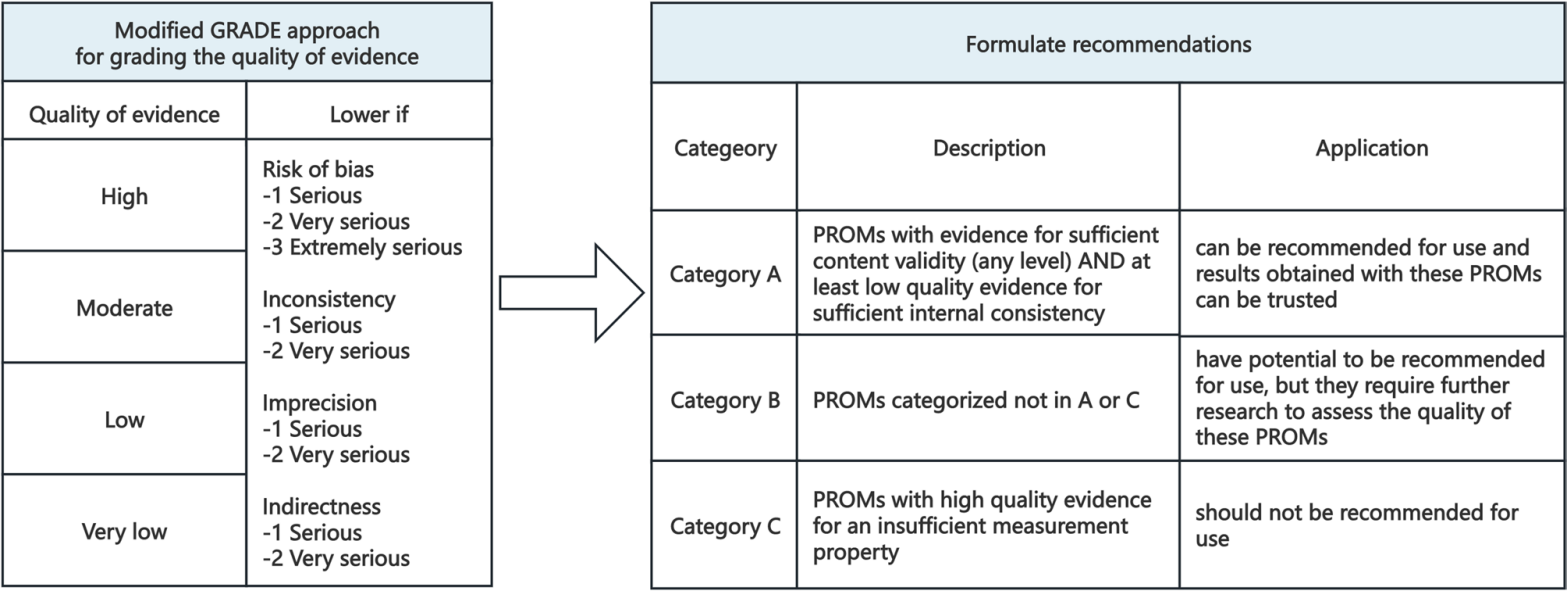
Modified GRADE approach for grading the quality of evidence and formulating recommendations for PROMs. (adapted from [18])

The modified Grading of Recommendations Assessment, Development and Evaluation (GRADE) was used to grade the certainty of evidence [18, 22]. The quality of evidence was initially rated as ‘high’ and then downgraded to ‘moderate’, ‘low’, or ‘very low’ based on risk of bias, inconsistency, imprecision, or indirectness. Based on the assessment results, the PROMs were categorized into three recommendation levels. When content validity is sufficient and internal consistency is sufficient (at least low-quality evidence), the instrument is classified as Category A and recommended for use. Category B recommendations are instruments that do not fit into categories A or C and that have potential for application but require more research to assess their quality. When there is high-quality evidence that any measurement property is insufficient, the instrument is classified as Category C and not recommended for use. The modified GRADE approach for grading the quality of evidence and formulating recommendations for PROMs is shown in Fig. 2 (adapted from [18]).

## Results

### Literature screening

**Fig. 3:**
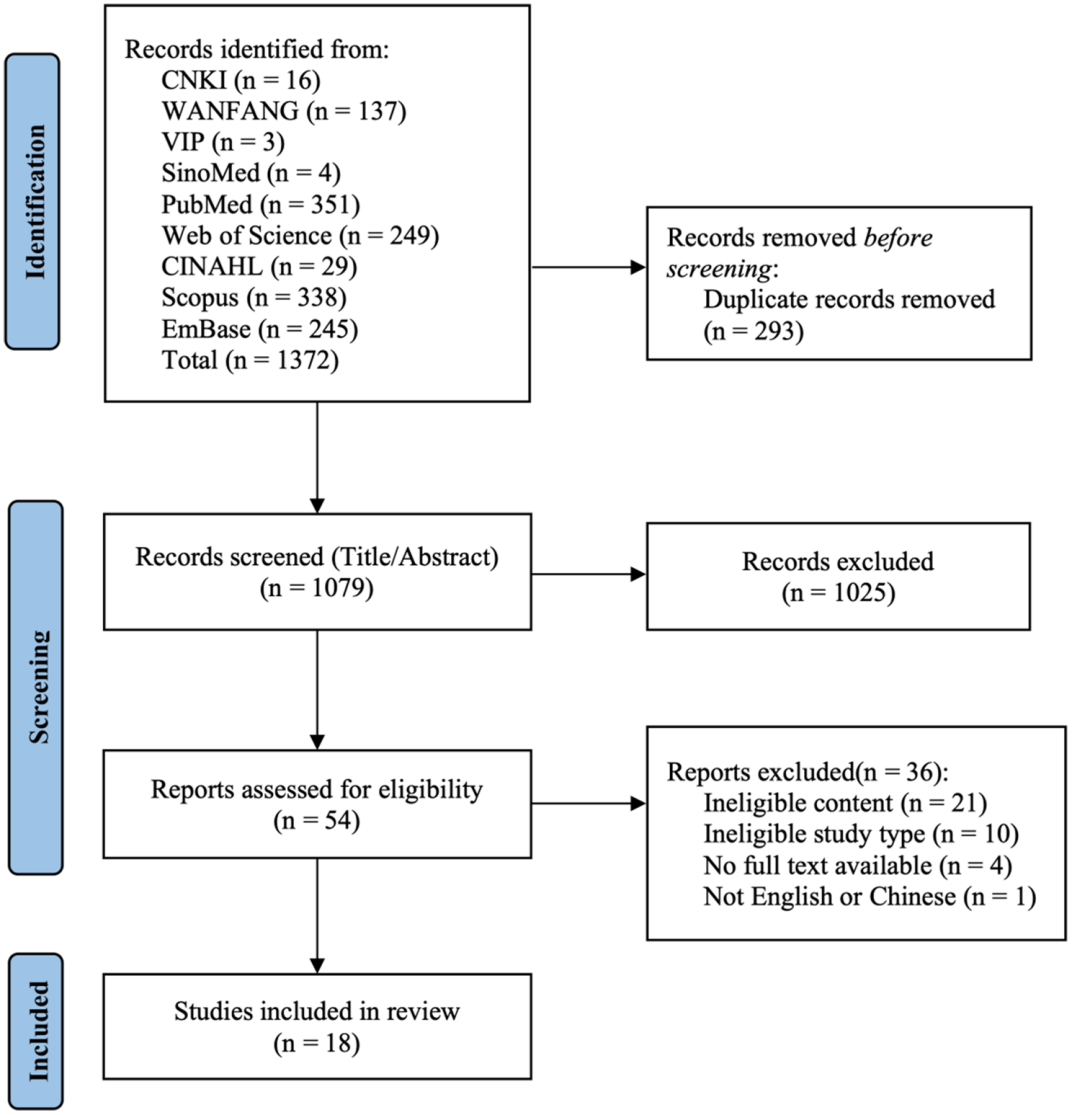
PRISMA flowchart for study screening and selection process

A total of 1,372 studies were obtained from the initial search of nine databases. After removing 293 duplicates, 1,025 studies were excluded based on titles and abstracts, and 36 studies were excluded after full-text review. Finally, a total of 18 articles were eligible for inclusion in this study. The PRISMA flow chart for the study screening process is depicted in Fig. 3.

### Study description

The 18 included studies involved eight PROMs, including COST [12, 23–30], PROFFIT [13, 31], FIT [14, 32], Financial Distress of Cancer Assessment Tool (FIAT) [33], Financial Toxicity Screening Measure (FinTox) [34], Subjective Financial Distress Questionnaire (SFDQ) [35], Hardship and Recovery with Distress Survey (HARDS) [36], and Financial Toxicity Measurement Tool for Breast Cancer Patients [37]. The characteristics of the 18 included studies are shown in Table 1.

**Table 1:** Basic characteristics of the included studies

First Author (Year)	PROM	Participant	Source	Country	Sample Size	No of Items and Domains	Measurement Domain	Total score range	Completion Time	Recall Period
de Souza [12] (2017)	COST	Advanced cancer patients	Cancer center	USA	233, 20	11/1	No subdomain	0–44 Likert5	NR	1 week
Yu [23] (2021)	COST-C	Cancer patients	Cancer hospital	China	440, 108	11/2	Positive wealth status, negative psychosocial response	0–44 Likert5	NR	2 weeks
Luo [24] (2023)	COST-C	Cancer patients	Cancer hospital	China	114, 22	11/3	Financial expenditure, financial resources, patient social response	0–44 Likert5	3–5 min	4 weeks
Liu [25] (2023)	COST-C	Older cancer patients	Hospital	China	205, 20	10/1	No subdomain	0–40 Likert5	NR	2 weeks
Wang [26] (2024)	COST-S-C	Cancer patients	Hospital	China	500	3/1	No subdomain	0–12 Likert5	NR	NR
Durber [27] (2021)	COST	Cancer patients	Hospital	Australia	257, 70	11/1	No subdomain	0–44 Likert5	NR	1 week
Kim [28] (2025)	COST-A	Cancer patients	Cancer center	Australia	122	13/2	NR	0–44 Likert5	NR	NR
Kajimoto [29] (2022)	COST-J	Gynecologic cancer patients	Cancer center and university hospital	Japan	112, 89	11/1	No subdomain	0–44 Likert5	NR	2–14 days
Shim [30] (2022)	COST-K	Breast cancer survivors	Medical center	Korea	4297, 202	11/1	No subdomain	0–44 Likert5	NR	1 week
Riva [13] (2021)	PROFFIT	Cancer patients	Oncological center	Italy	184, 132	16/1	No subdomain	0-100 Likert4	NR	3 weeks
Arenare [31] (2023)	PROFFIT	Cancer patients	Cancer center	Italy	221	16/1	No subdomain	0-100 Likert4	3–4 min	NR
Hueniken [14] (2020)	FIT	HNC patients	Cancer center	Canada	430, 19	9/3	Financial stress, financial strain, lost productivity	0-100 Yes/No, Likert3, Likert4, Likert5	NR	2 week ± 3 days
Jiang [32] (2023)	FIT-C	HNC patients	Cancer hospital	China	152, 50	9/3	Financial stress, financial strain, lost productivity	0-100 Yes/No, Likert3, Likert4, Likert5	NR	4 weeks
Richter [33] (2024)	FIAT	Cancer patients	Cancer center and hospital	Germany	378, 51–61	19/3	Financial worries, dissatisfaction across various life domains, challenging experiences with authorities and benefit providers	1–5 Likert5	NR	9–12 months
Badr [34] (2024)	FinTox	Cancer patients	Hospital	USA	268, 40	5/1	No subdomain	0–5 Yes/No	NR	1 week
Ahmad Dar [35] (2022)	SFDQ	HNC patients	Cancer center	India	142	14/3	Financial and psychosocial affect, coping behaviour, support seeking	0–28 Likert3	NR	NR
Liu [36] (2023)	HARDS	Older cancer patients	Hospital	China	518, 23	10/2	Subjective financial distress, objective medical burden	10–50 Yes/No, Likert5	5 min	2 weeks
Huang [37] (2024)	Financial Toxicity Measurement Tool for Breast Cancer Patients	Breast cancer patients	Hospital	China	300, 50	17/3	Subjective financial distress, objective financial burden, coping mechanism change	17–85 Likert5	2–3 min	2 weeks

Note: COST, Comprehensive Score for Financial Toxicity; COST-C, Chinese version of COST; COST-S-C, short Chinese version of COST; COST-J, Japanese version of COST; COST-K, Korean version of COST; PROFFIT, Patient-Reported Outcome for Fighting Financial Toxicity of cancer; FIT, Financial Index of Toxicity; FIT-C, Chinese version of FIT; FIAT, Financial Distress of Cancer Assessment Tool; FinTox, Financial Toxicity screening measure; SFDQ, Subjective Financial Distress Questionnaire; HARDS, Hardship And Recovery with Distress Survey; HNC, Head and Neck Cancer; NR, Not Reported

### Quality assessment

None of the 18 included studies [12–14, 23–37] evaluated measurement error. The assessment of methodological quality and measurement properties is presented in Table 2.

#### PROM development

Eight studies [12–14, 33–37] reported the PROM development. Four of these [12, 14, 34, 35] were rated as ‘doubtful’ for methodological quality due to unclear reporting on interviewer qualifications, data collection and analysis methods, and testing of instructions, items, response options, and recall period.

#### Content validity

Twelve studies [12–14, 24, 28, 30, 32–37] reported content validity. Seven of these [14, 24, 28, 30, 32, 34, 35] had unclear descriptions of data collection and analysis, resulting in a methodological quality rating of ‘doubtful’. The measurement properties of ten studies [12, 13, 24, 28, 30, 32–34, 36, 37] were rated as ‘sufficient’.

#### Structural validity

Sixteen studies [12–14, 23–26, 28, 30–37] reported structural validity. Six studies [23, 25, 28, 35–37] combined confirmatory factor analysis (CFA) and exploratory factor analysis (EFA), three studies [26, 31, 33] used CFA, and six studies [12–14, 24, 32, 34] used EFA. One study [30] used principal component analysis (PCA), with a methodological quality rating of ‘doubtful’. Four studies [14, 26, 30, 32] did not provide enough information, leading to an ‘indeterminate’ rating for measurement properties. One study [37] reported CFI < 0.95, and therefore, the measurement property was rated as ‘insufficient’.

#### Internal consistency

All 18 studies [12–14, 23–37] reported internal consistency with Cronbach’s α coefficients > 0.7. The methodological quality was rated as ‘very good’. Ten studies [14, 23–25, 27, 29, 30, 32, 35, 37] did not provide enough information for unidimensionality, resulting in an ‘indeterminate’ rating for measurement properties.

#### Cross-cultural validity

Two studies [12, 26] reported cross-cultural validity but were rated as ‘doubtful’ for methodological quality due to a lack of information on sample comparability. One study [12] conducted a differential item functioning (DIF) analysis but did not report the data, leading to an ‘indeterminate’ rating for measurement property.

#### Reliability

Fourteen studies [12–14, 23–25, 27, 29, 30, 32–34, 36, 37] reported reliability. Four studies [13, 32, 33, 37] did not clarify stability of measured construct, the rationale for retest intervals, or consistency in measurement settings, leading to a methodological quality rating of ‘doubtful’. One study [33] calculated Pearson correlation coefficients of 0.66, 0.64, and 0.75, leading to an ‘insufficient’ rating for measurement property.

#### Criterion validity

Seven studies [24, 31–34, 36, 37] reported criterion validity, using the COST or the financial difficulties subscale of EORTC QLQ-C30 as a reference. Four of these [31, 33, 34, 36] reported correlation coefficients < 0.7, resulting in measurement properties being rated as ‘insufficient’.

#### Hypotheses testing for construct validity

Fourteen studies [12, 14, 23, 24, 26–28, 30, 31, 33–37] reported hypothesis testing for construct validity. Three studies [35–37] calculated average variance extracted (AVE) and composite reliability (CR) values but did not provide detailed reports on hypothesized items, resulting in a methodological quality rating of ‘doubtful’. Five studies [24, 28, 35–37] had undefined or no hypotheses, leading to measurement properties rated as ‘indeterminate’.

#### Responsiveness

Two studies [14, 23] reported responsiveness, using paired t-test and Spearman correlation analyses to compare differences in scale scores before and after testing. The methodological quality of both studies was rated as ‘very good’. Due to the lack of clear hypotheses, their measurement properties were rated as ‘indeterminate’.

**Table 2:** Assessment of methodological quality and measurement properties of included studies

First Author (Year)	PROM	PROM Development	Content Validity			Structural Validity	Internal Consistency	Cross-Cultural Validity	Reliability	Criterion Validity	Hypotheses Testing	Responsiveness
			Relevance	Comprehensiveness	Comprehensibility							
de Souza [12] (2017)	COST	D	A^a^/+	NR	NR	A/+	V/+	D/?	A/+	NR	V/+	NR
Yu [23] (2021)	COST-C	NR	NR	NR	NR	V/+	V/?	NR	A/+	NR	V/+	D/?
Luo [24] (2023)	COST-C	NR	D^ab^/+	D^a^/+	D^a^/+	A/+	V/?	NR	A/+	V/+	V/?	NR
Liu [25] (2023)	COST-C	NR	NR	NR	NR	V/-	V/?	NR	A/+	NR	NR	NR
Wang [26] (2024)	COST-S-C	NR	NR	NR	NR	A/?	V/+	D/+	NR	NR	V/+	NR
Durber [27] (2021)	COST	NR	NR	NR	NR	NR	V/?	NR	A/+	NR	V/+	NR
Kim [28] (2025)	COST-A	NR	D^a^/+	D^a^/+	D^a^/+	V/+	V/+	NR	NR	NR	V/?	NR
Kajimoto [29] (2022)	COST-J	NR	NR	NR	NR	NR	V/?	NR	A/+	NR	NR	NR
Shim [30] (2022)	COST-K	NR	NR	NR	Da/+	D/?	V/?	NR	A/+	NR	V/+	NR
Riva [13] (2021)	PROFFIT	A	A^ab^/+	A^ab^/+	A^ab^/+	A/+	V/+	NR	D/+	NR	NR	NR
Arenare [31] (2023)	PROFFIT	NR	NR	NR	NR	V/+	V/+	NR	NR	V/-	V/+	NR
Hueniken [14] (2020)	FIT	D	D^b^/?	NR	NR	A/?	V/?	NR	A/+	NR	V/+	V/+
Jiang [32] (2023)	FIT-C	NR	D^b^/+	NR	D^ab^/+	A/?	V/?	NR	D/+	V/+	NR	NR
Richter [33] (2024)	FIAT	A	V^a^/+	NR	A^a^/+	V/+	V/+	NR	D/-	V/-	V/+	NR
Badr [34] (2024)	FinTox	D	D^ab^/+	D^a^/+	D^a^/+	A/+	V/+	NR	A/+	V/-	V/+	NR
Ahmad Dar [35] (2022)	SFDQ	D	D^ab^/?	D^a^/+	NR	V/+	V/?	NR	NR	NR	D/?	NR
Liu [36] (2023)	HARDS	V	A^ab^/+	V^b^/+	A^ab^/+	V/+	V/+	NR	A/+	V/-	D/?	NR
Huang [37] (2024)	Financial Toxicity Measurement Tool for Breast Cancer Patients	A	A^ab^/+	A^b^/+	A^ab^/+	V/-	V/?	NR	D/+	V/+	D/?	NR

Notes: The former (before ‘/’) indicates methodological quality; the latter (after ‘/’) indicates psychometric properties; V, Very good; A, Adequate; D, Doubtful; I, Inadequate; NR, Not Reported; +, Suffcient; -, Insuffcient; ?, Indeterminate; a, asking patients; b, asking professionals

### Certainty of evidence and recommendation

After assessing for risk of bias, inconsistency, and imprecision, the overall quality of evidence for each measurement property was determined. Based on these findings, the COST, FIT, and SFDQ were assigned a Category B recommendation. The PROFFIT, FIAT, FinTox, HARDS, and the Financial Toxicity Measurement Tool for Breast Cancer Patients were assigned a Category C recommendation. The detailed grading is presented in Table 3.

**Table 3:** Assessment of the level of inclusion in the study and recommendation

PROM	Content Validity		Structural Validity		Internal Consistency		Cross-Cultural Validity		Reliability		Criterion Validity		Hypothesis Testing		Responsiveness		Grade
	Rating	QOE	Rating	QOE	Rating	QOE	Rating	QOE	Rating	QOE	Rating	QOE	Rating	QOE	Rating	QOE	
COST	+	Moderate	+	Moderate	?	Moderate	+	Low	+	High	+	High	+	Moderate	?	Low	B
PROFFIT	+	Moderate	+	High	+	High	NR	NR	+	Low	-	High	+	High	NR	NR	C
FIT	+	Moderate	?	High	?	High	NR	NR	+	Low	+	High	+	High	+	High	B
FIAT	+	Moderate	+	High	+	High	NR	NR	-	Very Low	-	High	+	High	NR	NR	C
FinTox	+	Low	+	Moderate	+	High	NR	NR	+	Very Low	-	High	+	High	NR	NR	C
SFDQ	+	Low	+	High	?	High	NR	NR	NR	NR	NR	NR	?	Low	NR	NR	B
HARDS	+	High	+	High	+	High	NR	NR	+	Very Low	-	High	?	Low	NR	NR	C
Financial Toxicity Measurement Tool for Breast Cancer Patients	+	Moderate	-	High	?	High	NR	NR	+	Very Low	+	High	?	Low	NR	NR	C

Notes: QOE, Overall Quality of Evidence Grading; Grading QOE: High, Moderate, Low, Very Low

## Discussion

This systematic review, following the updated COSMIN guideline, evaluated the methodological quality and measurement properties of eight financial toxicity PROMs for cancer patients. The findings indicate that while several instruments show promise, the evidence on their measurement properties is incomplete or of limited quality, and consequently, no instrument met the criteria for a Category A recommendation. The COST emerged as the most thoroughly studied and promising tool, receiving a Category B recommendation. This review builds upon and updates previous systematic reviews in this area by applying the latest COSMIN guideline for a more contemporary and rigorous assessment.

### Incomplete reporting of development processes and methodological shortcomings

Content validity is the most important measurement property [38]. A transparent and rigorous development process is essential to achieving sufficient content validity. This process requires in-depth patient interviews to capture real experiences, combined with systematic literature reviews and expert consultations to define the construct being measured [39]. Four studies [12, 14, 34, 35] were rated as doubtful for their methodological quality in the development process. They briefly mentioned the involvement of patients and experts but did not report details. These reporting gaps diminish the evidence for content validity, subsequently undermining the scientific rigor and applicability of the financial toxicity measurement instruments. Future studies should strictly adhere to COSMIN reporting standards, adopt standardized qualitative research processes, and provide comprehensive documentation of the entire development process.

### Need for improved factor analysis and unidimensionality reporting

Structural validity of the included PROMs was frequently compromised by suboptimal methodological choices and incomplete reporting of key metrics [40]. Although confirmatory factor analysis (CFA) is preferred over EFA due to its rigor in hypothesis testing [18], half of the identified studies [12–14, 24, 30, 32, 34] used exploratory factor analysis (EFA) or principal component analysis (PCA), indicating certain methodological weaknesses. Moreover, key metrics such as the proportion of explained variance or factor loadings were frequently omitted, precluding a definite judgement and resulting in an indeterminate rating for structural validity. It is advised to first use EFA to explore the underlying factor structure, followed by CFA to confirm the model.

Unidimensionality is a prerequisite for a clear interpretation of the internal consistency parameter [18]. Ten studies [14, 23–25, 27, 29, 30, 32, 35, 37] did not clearly verify the unidimensionality of the PROMs, leading to an indeterminate rating for measurement properties. It is advised to first confirm the unidimensionality of the scale using factor analysis or IRT/Rasch methods, and then calculate the Cronbach’s α coefficient for (sub)scales to explain the correlations among items.

### Flawed retest design and weak evidence for criterion validity

Reliability requires that the construct remains stable between the repeated measurements and that measurement condition are the same [18]. Several studies failed to document these conditions and used Pearson correlation coefficients instead of the more appropriate ICC, weakening the accuracy of test-retest reliability. It is recommended to design measurement protocols rigorously, explicitly state the basis for construct stability and consistency of measurement conditions, select an appropriate time interval, and prioritize using ICC to evaluate test-retest reliability.

There is no gold standard for measuring financial toxicity in cancer patients currently. LEE suggested that in the absence of gold standard, instruments that are similar in measured construct or are multidimensional and comprehensive can be used as criteria [42]. Seven studies [24, 31–34, 36, 37] used the COST and financial difficulties subscale of the EORTC QLQ-C30 as the criteria. However, four studies [31, 33, 34, 36] showed weak correlations (< 0.7) with comparator instruments, indicating poor criterion validity. This deficiency limits the instruments’ utility for clinical screening and risk stratification. Researchers should select the most conceptually aligned instrument as a criterion and consider calculating metrics such as AUC, sensitivity, and specificity to determine the optimal cut-off values for each grade.

### Lack of predefined hypotheses for construct validity

Hypotheses testing examines whether PROM scores accord with theoretically predicted relationships. The more specific the hypotheses are and the more hypotheses are being tested, the more evidence is gathered for construct validity [18]. Five studies [24, 28, 35–37] did not propose hypotheses about the direction and strength of correlations or differences between groups, resulting in their measurement properties being rated as indeterminate. It is suggested to prespecify clear and theoretically-grounded hypotheses and provide thorough descriptions of comparator instruments, or the important characteristics of subgroups.

### Absence of cross-cultural validity, measurement error, and responsiveness testing

This review identified a significant lack of evidence for three critical measurement properties. While several instruments have been translated, the methodological rigor of these adaptations was often inadequate [18]. For instance, studies assessing cross-cultural validity [12, 26] failed to report information on sample or employ recommended statistical methods like multi-group CFA or DIF analysis. The gap affects the comparability of results across different cultural contexts. Measurement error was not evaluated in any of the included studies [12–14, 23–37], leaving it indeterminate whether a change in score reflects a true change in financial toxicity or merely measurement imprecision. Responsiveness reflects the PROM’s sensitivity to clinical changes. Sixteen studies [12, 13, 24–37] did not report responsiveness, limiting the instruments’ use in longitudinal monitoring and intervention evaluation. Future research should comprehensively evaluate these measurement properties, thereby improving the accuracy and reliability of PROMs.

### Content focus of included instruments and provisional recommendation for COST

Among the instruments receiving a Category B recommendation, the COST is provisionally recommended as the preferred tool for general use in assessing financial toxicity in cancer patients [11, 12]. This recommendation is based on it being the most extensively validated instrument across multiple studies and cultural contexts, with a comprehensive body of evidence supporting its measurement properties. Specifically, it demonstrated sufficient ratings for its content validity, structural validity, reliability, criterion validity, and hypotheses testing, supported by evidence ranging from moderate to high quality. It is suitable for rapid clinical screening (3–5 min) and available in 37 languages versions. In contrast, while FIT and SFDQ also showed promise, the evidence of FIT and SFDQ is more limited or context-specific. The FIT [14, 32] is designed for head and neck cancer patients and better addresses the unique challenges of this population, such as work limitations due to speech dysfunction. The SFDQ [35] is tailored for cancer patients undergoing radiation therapy and focuses on behavioral adjustments and psychological adaptation during the coping process. Therefore, FIT and SFDQ can be used as complementary measures for specific cancer.

### Implications for future research

Building on the evidence summarized in this review, several key implications for future research can be considered based on the COSMIN guideline. When developing new instruments, researchers should prioritize content validity from the outset. This requires conducting rigorous qualitative concept elicitation and pilot testing across diverse patient populations to ensure that the tool captures the comprehensive experience of financial toxicity. For existing instruments such as the COST—classified as Category B (provisionally recommended) in this review—systematic efforts are needed to address current evidence gaps. To advance the COST to Category A, future research should focus on three aspects: (1) strengthening the evidence base for content validity and internal consistency through well-designed studies in diverse populations, thereby consolidating the core requirements for Category A; (2) rigorously evaluating under-researched properties—specifically measurement error, cross-cultural validity, and responsiveness—given that these were identified as critical evidence gaps in this review; and (3) applying rigorous study designs and appropriate statistical methods with pre-specified hypotheses for construct validity, to address common methodological limitations observed in the included studies. Future validation studies should adhere to the COSMIN reporting standards for clear and complete documentation of study design, methods, and results. By generating high-quality evidence across these areas, future research can contribute to ensuring that instruments used in clinical practice and research are scientifically sound and relevant to patients. This may ultimately support better identification of at-risk individuals, more effective targeting of interventions, and progress toward reducing financial disparities and improving well-being in cancer patients.

### Limitations

There are some limitations to this study. First, some COSMIN criteria involve subjective judgment, which could introduce bias. Secondly, only Chinese and English publications were included, thereby excluding potentially relevant studies in other languages. Thirdly, the measurement properties of some instruments were assessed by only a single study, which may affect the reliability of the findings.

## Conclusion

This study followed the COSMIN guideline to comprehensively evaluate measurement instruments for financial toxicity in cancer patients. It found that their methodological quality and measurement properties still require significant improvement. Based on the available evidence, the COST is provisionally recommended and can be supplemented with other instruments. The findings highlight the need for further high-quality research to address critical evidence gaps and to strengthen the evidence base for core measurement properties such as content validity and internal consistency. Future research should rigorously adhere to COSMIN guideline to facilitate the development and validation of instruments that are both scientifically sound and clinically meaningful for cancer patients. 

## Data Availability

All data generated or analyzed during this study are included in this published article.
